# Mapping the path to recovery: the intersection of cortical thickness reductions and serotonin transporter expression in anorexia nervosa

**DOI:** 10.1038/s41380-025-03306-4

**Published:** 2025-11-29

**Authors:** Livio Tarchi, Arne Doose, Fabio Bernardoni, Joseph A. King, Inger Hellerhoff, Klaas Bahnsen, Andreas Hess, Arnd Dörfler, Stefanie Horndasch, Giovanni Castellini, Valdo Ricca, Michael Marxen, Veit Roessner, Paul M. Thompson, Stefan Ehrlich

**Affiliations:** 1 Psychiatry Unit, Department of Health Sciences, University of Florence, Florence, Italy; 2 Division of Psychological and Social Medicine and Developmental Neurosciences, Translational Developmental Neuroscience Section, Faculty of Medicine, Technische Universität Dresden, Dresden, Germany; 3 German Center for Child and Adolescent Health (DZKJ), partner site Dresden/Leipzig, Dresden, Germany; 4 Department of Neuroradiology, University Clinic Erlangen, Erlangen, Germany; 5 Institute for Pharmacology and Toxicology, University Erlangen-Nuremberg, Erlangen, Germany; 6 Department of Child and Adolescent Psychiatry, University Clinic Erlangen, Erlangen, Germany; 7 Department of Psychiatry and Psychotherapy, Carl Gustav Carus University Hospital, Technische Universität Dresden, Dresden, Germany; 8 Department of Child and Adolescent Psychiatry, Faculty of Medicine, Technische Universität Dresden, Dresden, Germany; 9 Imaging Genetics Center, Stevens Neuroimaging & Informatics Institute, Keck School of Medicine, University of Southern California, Los Angeles, USA; 10 Department of Child and Adolescent Psychiatry, Eating Disorder Research and Treatment Center, Faculty of Medicine, Technische Universität Dresden, Dresden, Germany

**Keywords:** Neuroscience, Predictive markers

## Abstract

Severe reductions in the brain’s cortical gray matter thickness (CT) have repeatedly been reported in anorexia nervosa (AN). The underlying mechanisms of these drastic changes remain unclear. Associations with potential underlying neurochemical and metabolic features have not been evaluated. In this study, we investigated whether CT alterations, across the cortical surface, in AN (n = 114) compared to healthy controls (HC; n = 114 age-matched, range 12–29 years old) might be associated with the spatial distribution of neurotransmitter receptors, transporters and/or metabolic glucose uptake (i.e., “chemoarchitecture”, based on data from PET binding studies in healthy individuals, as implemented by *neuromaps*). First, the correlation between CT alterations in AN and chemoarchitecture was evaluated at the group-level. Second, chemoarchitecture was leveraged to compute per-participant correlations of neuroreceptor maps with CT alterations at the individual-level. Correlations with psychiatric symptoms and associations with early weight gain (30-days after admission) were tested. Group-level results were replicated in an external sample. Regions showing substantial cortical thinning in AN were also characterized by higher AChN receptor density and higher glucose metabolism uptake. While CT was comparatively preserved in AN at the group-level in regions with higher HT1a and SERT receptor density, individual participants who exhibited cortical thinning in these regions also reported more severe symptoms (depression and body dissatisfaction) and showed less treatment-related weight gain. These associations may help to define a biological risk signature for AN, possibly allowing for future applications in treatment stratification and/or personalization.

## Introduction

Anorexia Nervosa (AN) is a life-threatening eating disorder, with a typical onset during adolescence. AN is characterized by an intense fear of weight gain and body image dissatisfaction, restriction of energy intake, low weight, and additional symptoms such as increased physical activity [1]. Currently, no approved medications for the treatment of AN exist [2]. Intensive nutritional and psychological treatment for AN requires long hospital stays, has a response rate of only ~ 50%, and up to one third of affected individuals may become chronically affected [3].

Despite the increasing prevalence of AN and the significant burden on those affected and their caregivers [4], a comprehensive understanding of the disease’s pathophysiological mechanisms remains elusive. Reductions of the brain’s gray matter have long been reported in AN [5], evidenced by meta-analyses integrating diverse international datasets [6]. Cortical thickness (CT) has been shown to be consistently reduced in the acutely underweight state of AN, representing a hallmark of the disorder [7]. The magnitude of cortical thinning in AN is the largest among all psychiatric disorders [6, 7]. Although cortical thinning is widespread and pronounced, it seems to rapidly reverse after weight restoration therapy [7–9].

The underlying mechanisms of these dynamic changes in CT remain poorly understood, but are of critical interest for understanding therapeutic effects. Previously, associations with the brain connectome in terms of hub structure were described [6], as well as the involvement of specific cell types by means of virtual histology [7]. However, associations with potential underlying neurochemical and metabolic features have not been evaluated. In this regard, “data enrichment” refers to techniques that allow to enhance one’s own measurements using additional information from different sources. Novel tools have emerged which help to examine the association between specific neuroimaging features, and the underlying spatial distribution of neurotransmitter receptors, transporters or metabolic signatures in the central nervous system [10, 11]. The spatial distribution of these signatures has been referred to as the underlying “chemoarchitecture” of the brain [10].

Here we examine whether the brain’s chemoarchitecture and its metabolism may be spatially aligned with the pattern of CT alterations commonly detected in acutely underweight AN patients. This enriched characterization of CT alterations might help to identify biological targets for personalized interventions in AN [5]. In wake of the COVID-19 pandemic, AN incidence is rising [12], and healthcare services at large are impacted by an increasing number of patients not receiving adequate treatment [13]. In the light of these significant challenges, scientists in the field are eager to identify predictors of illness trajectories and treatment outcomes for patients with AN [14].

Prior work suggests that a multivariate signature of individual-level CT alterations identified by machine learning may be able to predict one-year outcome [15]. The identification of specific CT alterations – spatially aligned with discrete aspects of the brain’s chemoarchitecture – may also reveal information that is closer to the biological mechanisms and thus provide more precise outcome predictions at the individual level in patients with AN than those related to aggregate regional measures. Therefore, in the current work, we focused on predicting early weight gain, as this in turn has been shown to be a significant predictor of long-term recovery [16, 17]. By focusing on early identification of patients at increased risk of poorer outcomes, healthcare providers can tailor individualized treatment approaches and potentially optimize resource allocation.

### Aims

The primary aim of the study was to investigate how the spatial distribution of vertex-wise CT alterations in acutely underweight patients with AN is related to the underlying chemoarchitecture and metabolic situation of the brain. As exposure to radioactive tracers poses significant clinical and ethical concerns in acutely underweight patients, and as PET data (with whole brain coverage) obtained in AN are scarce—–the present study leveraged previously collected PET data in healthy individuals as implemented by neuromaps [11]. First, group differences in CT between acutely underweight AN and healthy controls (HC) were estimated. The resulting group contrast was evaluated in relation to the underlying chemoarchitecture. Leveraging previously collected data within the ENIGMA Eating Disorders working group [6], we replicated our main results on an external sample.

The secondary aims of the study were: first, to investigate whether individual-level CT alterations, at the vertex-level, were correlated with the spatial distributions of chemoarchitecture, and whether this individual-level correlation would explain individual symptom severity. Second, we tested whether this individual-level correlation between CT alterations and chemoarchitecture was predictive of early weight gain during intensive weight rehabilitation treatment (beyond the known effects of BMI-SDS at baseline alone).

## Materials and methods

### Sample and participants

Our primary study cohort was a pairwise age-matched case-control sample, [7] consisting of 114 acutely underweight, predominately non-chronic participants with AN (mean age: 16.57 years +/− 3.31 SD; mean duration of illness: 13.8 months  +/− 19.89 SD), and 114 HCs (mean age: 16.63 years +/− 3.34 SD; age-range 12–29 years old) derived from previous work [7]. Participants suffering from AN were assessed within 96 h of admission to an intensive behaviorally-oriented nutritional treatment program at the specialized eating disorder programs at Child and Adolescent Psychiatry and Psychosomatic Medicine Departments of the University Hospital Dresden. For more details see Supplementary Information, Methods S1. The study protocols were ethically approved by the local Institutional Review Board of the Technische Universität Dresden, and all participants (and, if minors, their legal guardians) gave written informed consent.

BMI, sex and age-corrected BMI-standard deviation score (BMI-SDS) [18, 19] of patients were measured at the day of scanning and 30 days after admission to the inpatient rehabilitation program (lost to follow-up, n = 21). We used the expert form of the Structured Interview for Anorexia and Bulimia Nervosa (SIAB-EX [20] and the Eating Disorder Inventory-2 (EDI-2) [21] were adopted to assess eating disorder-related psychopathology. Depressive symptoms were assessed using the Beck Depression Inventory-II (BDI-II) [22]. As in prior reports [23, 24], we focused our analyses on “core” eating disorder domains, namely the “drive for thinness”, “body dissatisfaction”, and “bulimia” subscales of the EDI-2. Study data were managed using Research Electronic Data Capture (REDCap) [25].

The replication sample comprised data from AN (N = 21) patients and HC (N = 28) collected by our collaborators via the ENIGMA Eating Disorders working group in Erlangen, Germany, and has been part of previous publications [6, 26, 27]. For a detailed sample description and methods, see Supplementary Information, Table S1 and Supplementary Information, Methods S4 respectively.

### Image acquisition and analysis

The current study was performed on a 3 T Magnetom Trio Scanner (Siemens Healthineers, Erlangen, Germany) with a 32-channel head coil. All scans were collected between 8 and 9 a.m., to reduce the impact of time of day on brain morphometric measures [28]. 3D T1-weighted volumetric brain images were acquired using a magnetization-prepared rapid gradient-echo (MP-RAGE) sequence, with the same parameters as in previous studies [7]. CT was calculated for each vertex, and mean global values annotated, in accordance with previous studies [7]. See Supplementary Information, Methods S2, for further details.

### Group contrast

FreeSurfers’ general linear model was used to calculate the statistical difference between AN and HC for vertex-wise CT [29], with age as a covariate (see Eq. 1), where the second linear term represents the group contrast.

1 $$(\overline{{CT}})={\beta }_{0}+{\beta }_{1}* {age}+{{\beta }_{2}}_{({group})}+e$$

### Individual-level CT alterations

To assess the individual-level CT alterations, subject-level residuals (term *e* in Eq. 1) were first extracted and then employed for data enrichment. Subject-level residual errors were defined as the difference from the vertex-wise predicted CT value, as a function of age. This individual-level residual was interpreted as a functional index of deviation from age-predicted CT.

### Chemoarchitecture - reference feature maps

The Python package *neuromaps* [11] is a collection of methods and maps that allows for comparison of empirically measured maps (e.g., CT) with a number of reference feature maps. The reference feature maps are derived from PET scans of healthy individuals [10, 11], and the associated neurochemicals have been previously investigated in AN [30–39]. The neuromaps package has been utilized in the study of the alignment of structural alterations with the chemoarchitectural features of the brain, both at the group and individual levels [40, 41]. The following reference feature maps, previously obtained in healthy individuals, were retrieved [10, 11]: acetylcholine receptor (both nicotinic - AChN, and muscarinic - AChM1), dopamine transporter (DAT) and dopamine receptor (D1 and D2), serotonin transporter (SERT) and serotonin receptors (HT1a, HT1b, HT2a), norepinephrine transporter (NET), histamine receptor (H3), opioid receptor (MOR), cannabinoid receptor (CB1), GABA receptors (GABA), glutamate receptors (Glut), glucose metabolism (Glc, representing the rate of aerobic glycolysis of the brain in response to cognitive load). In total, sixteen reference feature maps were used to test correlations between CT alterations and chemoarchitecture at the group level. For more details, see Methods S3 in the Supplementary Information.

### Statistical analysis

#### Spatial enrichment of group-level CT alterations

To enrich group-level CT alterations with the reference feature map of choice, *neuromaps* was employed in order to transform data to the native space of choice (*fsaverage*). In brief, as previously mentioned, *neuromaps* uses standard tools [42–45] to perform the transformation between surfaces, volumes or from volumes to surfaces. We used surface-to-surface transformation to convert the reference feature maps to the *fsaverage* map. Once transformed to this common space, *neuromaps* was used to estimate the vertex-wise spatial correlation (Pearson) between the two images across the whole surface [11]. To determine whether the measured correlations were statistically significant, null models were computed by rotating the reference images (n = 5000), thus deriving the null distribution of correlation coefficients [46].

#### Spatial enrichment of individual-level CT alterations

Enrichment of individual-level CT alterations was performed by comparing the individual-level residuals introduced above (Eq. 1 – term e), representing the deviation from age-predicted CT with reference feature maps. Similarly to the spatial enrichment of group-level CT alterations, *neuromaps* was here used to estimate the vertex-wise spatial correlation (Pearson) between the two images across the whole surface [11] and at the individual level. A correlation coefficient per-participant, per-map was thus estimated.

#### Clinical correlates of spatially enriched individual-level CT alterations

Spatially enriched individual-level CT alterations in participants with AN were then correlated with depressive (BDI II total score) and eating psychopathology (EDI-2, both its total score, and the three core domains previously described), using age and BMI-SDS adjusted Spearman rank coefficients (rho). Finally, to account for multiple comparisons when correlating spatially enriched individual-level CT alterations with the symptom scores (as described above), the statistical significance of the results was adjusted using the Benjamini-Hochberg [47] procedure (row-wise, correcting for five comparisons across four neurochemical features), and the False Discovery Rate (FDR, p < 0.05) was computed. In a second iteration, as adjusting for BMI-SDS could mask correlations with eating psychopathology, partial Spearman coefficients were only adjusted for age.

#### Individual predictors of weight restoration

Linear regression models were then used to predict weight restoration after 30 days of intensive treatment. The BMI-SDS at baseline was subtracted from the BMI-SDS at 30 days, in order to compute the ΔBMI-SDS. As prior evidence has shown that BMI-SDS restoration in patients with AN can be better appreciated as a function of relative rather than absolute weight change, BMI-SDS at baseline was used as an additional predictor [48].

2 $$\,{\varDelta {BMI}}_{{SDS}} = 	 \,{\beta }_{0}+{\beta }_{1}* {age}+{\beta }_{2}{{{BMI}}_{{SDS}}}_{{baseline}}+{\beta }_{3}{speCT} \\ 	 +{\beta }_{4}({{{BMI}}_{{SDS}}}_{{baseline}}* {speCT})$$

In Eq. 2, the term *speCT* represents the spatially enriched individual-level CT alterations (see previous section for further details).

As individual-level CT alterations are strongly correlated with BMI-SDS [8], the interaction term between BMI-SDS and spatially enriched individual-level CT alterations allows for the joint estimation of the effect attributable to CT alterations alone, and that of the interaction term between CT alterations and BMI-SDS. This interaction term evaluates possible moderating effects played by spatially enriched individual-level CT alterations on the known relationship between low BMI-SDS and less successful longitudinal weight restoration [16].

To probe this interaction term, the Johnson-Neyman interval was computed for statistically significant interaction terms [49]. The Johnson-Neyman interval determines the range within which the moderation effect is significant, indicating whether this effect is homogeneous across the observed data [50]. Heteroskedasticity-consistent (HC3) standard errors were used to estimate confidence intervals within the Johnson-Neyman technique [51].

#### Control analyses

Additional linear regression models of clinical outcome were estimated, to assess the specificity of the findings for spatially enriched individual-level CT alterations. These models were computed with global CT as a predictor (Alternative Model 1), as a covariate (Alternative Model 2), or as an interaction term (Alternative Model 3 and 4). These models were computed to verify whether the effect observed for spatially enriched CT alterations depended on their spatial distribution (and thus, the underlying feature), or global thickness reduction.

Furthermore, the main model for individual predictions of weight restoration (Eq. 2) was recalculated in subsamples where we excluded participants with possible confounding characteristics; i.e., restricting the sample to patients within the restrictive subtype alone, without psychiatric comorbidities, and without a recent exposure to antidepressants. See Table 1 for sample details.

**Table 1:** Sample Descriptives.

	AN	HC	T-score	p-value
	(n = 114)	(n = 114)		
Demographics				
Age (years ± SD)	16.57 ± 3.31	16.63 ± 3.34	0.137	0.891
Minimum lifetime BMI (kg/m^2^)	14.43 ± 1.36	19.43 ± 1.88	22.033	<0.001
BMI-SDS at baseline	−3.20 ± 1.27	−0.085 ± 0.67	23.185	<0.001
BMI-SDS at 30 days	−2.15 ± 0.91	/	/	/
BMI-SDS at 60 days	−1.26 ± 0.75	/	/	/
Psychometrics				
BDI-II total score	22.76 ± 11.08	5.51 ± 5.50	−14.838	<0.001
EDI_DT	28.99 ± 9.11	13.67 ± 0.56	−14.979	<0.001
EDI_BD	37.21 ± 10.57	23.27 ± 9.30	−10.526	<0.001
EDI_B	11.11 ± 4.98	9.78 ± 2.89	−2.449	0.015
EDI total score	210.57 ± 45.25	140.35 ± 29.25	−13.729	<0.001

Mean value ± standard deviation (SD) for each variable and study group are shown. Group differences were tested using Welch two sample t-tests. As test statistics, t-value, and p-values are stated.

Restrictive subtype was diagnosed in 102 AN (89.5%) and binge/purge subtype in 12 (10.5%). Eighteen patients had other psychiatric comorbidities. Four patients had a recent exposure to antidepressant drugs (prior 14 days).

*AN* anorexia nervosa, *HC* healthy controls, *BDI-II* beck depression inventory–II, *BMI-SDS* body mass index-standard deviation score, *EDI* eating disorder inventory–2, *DT* drive for thinness, *BD* body dissatisfaction, *B* bulimia.

## Results

The AN and HC groups did not differ in age. As expected, AN patients had markedly lower BMI-SDS in comparison to controls. EDI core symptoms (drive for thinness, body dissatisfaction, bulimia), and BDI total scores were markedly higher in AN than HC (see Table 1).

### Spatial enrichment of group-level CT alterations

**Fig. 1:**
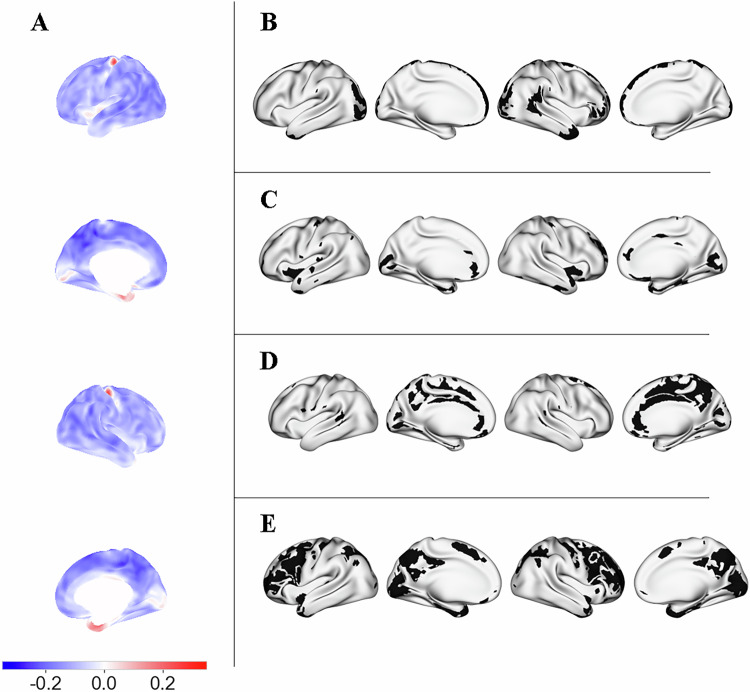
Spatial enrichment of group-level cortical thickness (CT) alterations in anorexia nervosa (AN), depicting CT alterations in AN and relevant intersection with chemoarchitecture density maps. Left column (**A**): Whole-brain CT alterations in individuals with AN compared to healthy controls. Color scale: contrast map (in cm) of the group comparison—blue indicates lower CT in AN, and red indicates higher CT in AN; Right column (**B,** **C,** **D,** **E**): Regions with marked CT differences were defined as those showing a group difference of ±0.1 cm. Chemoarchitectural feature maps were thresholded at 1 standard deviation (SD) from the mean (except for Glc, which was thresholded at 0.5 SD to enhance visual clarity). Intersected regions were further thresholded to a minimum cluster size of 15 vertices. The most prominent resulting intersections are displayed to facilitate visual comparison. Overlays of CT and chemoarchitecture maps can be found in Supplementary Fig. S2. **B** AChN, Acetylcholine nicotinic receptor*;* **C**: HT1a, serotonin receptor; **D** SERT, Serotonin transporter; **E**: Glc, glucose metabolism.

As in prior analyses [7], widespread cortical thinning was detected in AN relative to HC (except for three regions, which did not significantly differ between AN and HC, namely: the inferior aspect of the cingulate gyrus, superior insular lobes, the cuneus/pericalcarine gyrus). Two regions were significantly reduced in HC in comparison to AN (the medial aspect of the precentral gyrus, the anterior pole of the inferior temporal lobe; for detailed results see Bahnsen et al., Fig. 1a).

**Fig. 2:**
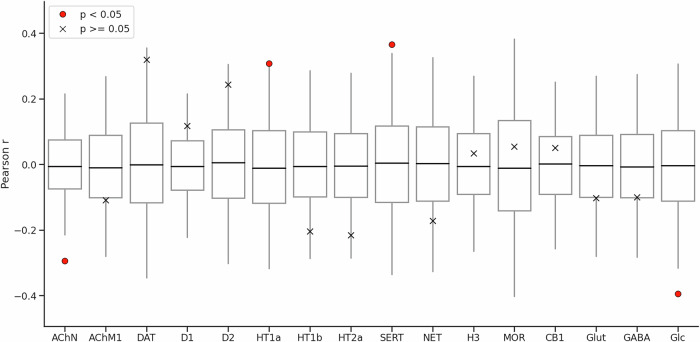
Spatial enrichment of group-level cortical thickness alterations in anorexia nervosa (AN), compared to age- and sex-matched healthy controls addressing the primary aim of this study to investigate whether the spatial distribution of vertex-wise CT alterations in acute AN is related to the underlying chemoarchitecture. Boxplots show the correlation coefficients for rotated images (5000 permutations), to represent 95% confidence intervals of null distributions [46]. A cross denotes non-significance; a red dot denotes p < 0.05, FDR corrected for multiple comparisons. AChN, Acetylcholine nicotinic receptor; AChM1, Acetylcholine muscarinic receptor; DAT, dopamine transporter; D1/D2, Dopamine receptors; HT1a, HT1b, HT2a, serotonin receptors; SERT, Serotonin transporter; NET, norepinephrine transporter; H3, histamine receptor; MOR, opioid receptor; CB1, cannabinoid receptor; Glut, glutamate receptor; GABA, GABA receptor; Glc, glucose metabolism.

The distribution of CT reduction in AN showed significant correlation with reference features derived from a range of previous PET studies conducted in healthy individuals (Fig. 2). In particular, the CT group contrast map was significantly correlated with the spatial distribution of the following feature maps: AChN (r = −0.294, FDR p = 0.003; most notably in midline frontal and parietal regions, bilateral temporal pole, right fronto-lateral regions, right gyrus angularis, bilateral latero-occipital regions, Fig. 1b, Supplementary Information, Fig. S2), HT1a receptors (r = 0.308, FDR p = 0.033; most notably in bilateral medial anterior lobes, bilateral medial occipital regions, bilateral antero-medial temporal lobes, bilateral inferior frontal cortex, bilateral antero-frontal cingulate cortex, Fig. 1c, Supplementary Information, Fig. S2), SERT (r = 0.366, FDR p = 0.003; most notably in bilateral cingulate cortex, bilateral precuneus, bilateral medial occipital regions, Fig. 1d, Supplementary Information, Fig. S2), glucose metabolism (r = −0.394, FDR p = 0.024; most notably in bilateral fronto-parietal regions, bilateral inferior temporal lobes; Fig. 1e, Supplementary Information, Fig. S2).

### Clinical correlates of spatially enriched individual-level CT alterations

The correlation between spatially enriched individual-level CT alterations and symptom scores was also evaluated. Only feature maps exhibiting significant correlations with CT alterations at the group-level were included (AChN, HT1a, SERT, Glc).

**Fig. 3:**
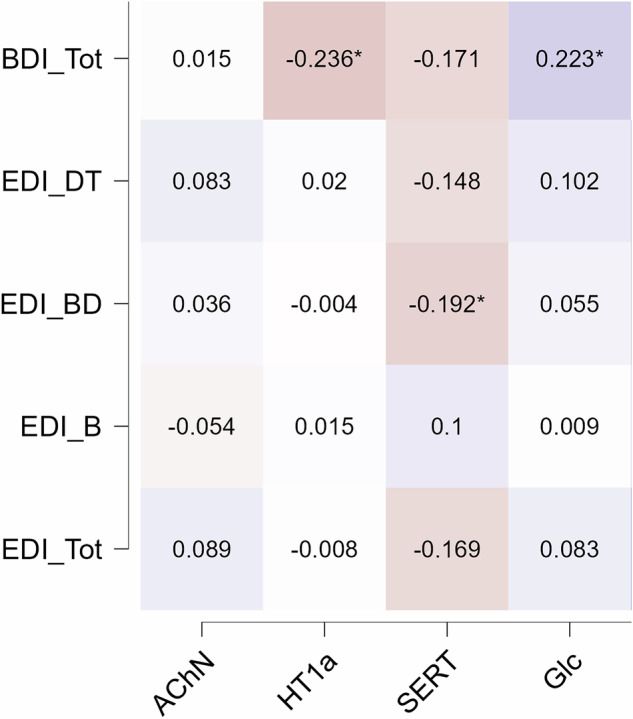
Spatial enrichment of individual-level cortical thickness alterations, correlations with clinical scores. We examined whether the individual-level correlation of cortical thickness alterations with the spatial distributions of chemoarchitecture was correlated with clinical symptom severity. BDI-II associations with HT1a and SERT survived FDR correction for multiple comparisons (adjusted p < 0.05), while the association between EDI-2 Body Dissatisfaction and SERT did not. Spearman rho coefficients were corrected for both age and BMI-SDS. Results were computed only within patients. In red negative correlations; in blue positive correlations. Lighter colors indicate weaker correlation, darker colors indicate stronger correlation. BDI-II beck depression inventory, EDI-2 eating disorder inventory 2, DT drive for thinness, BD body dissatisfaction, B Bulimia.

Three maps of spatially enriched CT alterations were significantly associated with psychopathology, beyond effects of BMI-SDS and age (Fig. 3). These significant correlations were between BDI total score and HT1a (partial rho = −0.236, p = 0.012, FDR p = 0.036) and Glc (partial rho = 0.223, p = 0.018, FDR p = 0.036). One statistical association, between body dissatisfaction and SERT (partial rho = −0.192, p = 0.043), did not survive multiple comparisons correction (FDR p = 0.172).

### Individual predictors of clinical outcomes

Next, spatially enriched individual-level CT alterations were tested as predictors of ∆BMI-SDS after 30 days of intensive treatment. Again, only feature maps exhibiting significant correlations with CT alterations at the group-level were included (AChN, HT1a, SERT, Glc). According to the results obtained using Eq. 2, SERT significantly predicted BMI-SDS after 30 days of intensive treatment. Specifically, patients with greater cortical thinning in regions associated with higher SERT density had less treatment-related weight gain. Furthermore, SERT moderated the predictive effect of BMI-SDS at baseline. This moderation term can be interpreted as lower BMI-SDS at baseline predicting even less successful longitudinal weight restoration if SERT-associated brain regions were affected by CT reductions. In other words, although CT was comparatively preserved in regions with high SERT expression at the group level (Fig. 1, Fig. 2), individual patients who exhibited comparatively more cortical thinning in SERT-associated brain regions were more likely to experience less successful short-term weight restoration (Table 2)—even more so if they had an exceptionally low BMI.

**Table 2:** Impact of spatially enriched individual-level CT alterations on ΔBMI-SDS recovery at 30 days.

	Main Effect (Spatially enriched CT Alterations)	Moderation term (interaction with BMI-SDS at baseline)
AChN	0.512 (p = 0.475)	0.270 (p = 0.189)
HT1a	−0.477 (p = 0.509)	−0.067 (p = 0.752)
SERT	−1.592 (p = 0.019)*	−0.524 (p = 0.010)*
Glc	−0.413 (p = 0.577)	−0.104 (p = 0.645)

Linear model corrected for the effect of weight and age at baseline.

*CT* cortical thickness, *BMI-SDS* body mass index-standard deviation score, *AChN* acetylcholine nicotinic receptor, *HT1a* serotonin receptor, *SERT* serotonin transporter, *Glc* glucose metabolism.

*significant at p < 0.05.

**Fig. 4:**
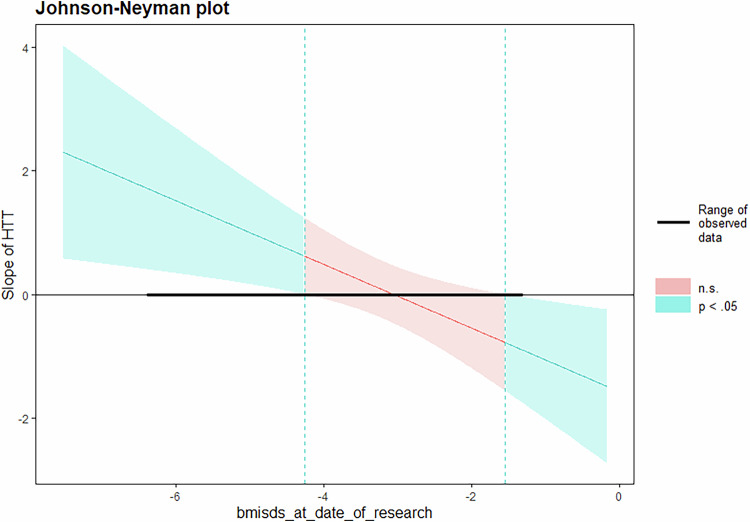
Individual-level cortical thickness alterations in areas expressing SERT impact weight restoration only once a certain severity threshold is reached. As part of our secondary objectives, we investigated whether alterations in CT at the individual level exhibited a correlation with chemoarchitecture and could serve as a predictor of early weight gain during intensive weight rehabilitation treatment. Moderation effect of weight reduction at baseline on the main effect of SERT, for predicting weight restoration after 30 days of intensive treatment. On the y-axis, the slope of SERT. On the x-axis, BMI-SDS. n.s. non-significant.

The correlation analysis using the Johnson-Neyman technique revealed a significant overall interaction. The interaction became especially pronounced within the interval of BMI-SDS [-inf, −4.67] and [−1.96, + inf]. The boundaries of significance for the moderation term were: positive at −1 SD of BMI-SDS (β = 0.830, p = 0.05); negative at + 1 SD (β = −0.560, p = 0.05, Fig. 4).

As 16 individuals were included in the sample for the range of BMI-SDS between 0 and −2, this analysis was repeated including only patients with a BMI-SDS below −2. This analysis confirmed that the boundaries of significance were within the interval BMI-SDS of [-inf, −4.37] (p < 0.05, Supplementary Information, Fig. S3).

### Control analysis - confounding effect of global CT

In Alternative Model 1, which evaluated the effect of global CT, global CT did not predict weight restoration after 30 days of intensive treatment, thus confirming that enriched CT alterations and not cortical thinning per se drove the observed moderation effect.

In Alternative Model 2, the main effect of global CT was assessed in its role as a confounder, in addition to the standard model of SERT and SERT interaction with BMI-SDS at baseline. In Alternative Model 3 and 4, both the main effect and the interaction terms were calculated to further control for confounders. In brief, the alternative models confirmed the specificity of previous findings, and showed no significant confounding effect of global CT in estimating weight restoration in AN after 30 days of intensive treatment (Table 3).

**Table 3:** Control Analyses, the effect of SERT on ΔBMI-SDS restoration at 30 days.

	SERT Main Effect	SERT Moderation term (interaction with BMI-SDS at baseline)	Global CT Main Effect	Global CT Moderation term (interaction with SERT)	Global CT Moderation term (interaction with BMI-SDS at baseline)
Alternative Model 1	/	/	0.573 (p = 0.441)	/	0.259 (p = 0.281)
Alternative Model 2	−1.713 * (p = 0.013)	−0.540 * (p = 0.007)	−0.268 (p = 0.349)	/	/
Alternative Model 3	−5.519 (p = 0.392)	−0.568 * (p = 0.006)	−0.343 (p = 0.275)	1.489 (p = 0.553)	/
Alternative Model 4	−6.253 (p = 0.339)	−0.549 * (p = 0.008)	0.200 (p = 0.790)	1.812 (p = 0.477)	0.192 (p = 0.426)

Linear model corrected for the effects of weight and age at baseline, predicting ΔBMI-SDS, after 30 days of intensive treatment.

*CT* cortical thickness, *BMI-SDS* body mass index-standard deviation score, *SERT* Serotonin transporter.

*significant at p < 0.05.

### Control analysis - subsample analysis

Individual-level CT alterations spatially enriched for SERT negatively predicted ∆BMI-SDS after 30 days of intensive treatment also when restricting the analyses to smaller subsamples of individuals including e.g. only the patient cohort with the restrictive AN subtype (Supplementary Information Table S2, Figure S3). Also, the interaction term between SERT enriched individual-level CT alterations and BMI-SDS at baseline was predictive of individual weight restoration in patients, for all subsample analyses.

### Control analysis - out of sample replication

The CT group contrast map in the independent dataset showed a similar pattern of global CT reductions in AN compared to HC (Supplementary Information, Fig. S1a and Figure S1b). We were able to partially replicate the correlation of the distribution of CT reduction in AN with reference features. In particular, the group contrast map of the replication sample was correlated with the spatial distribution of AChN (r = −0.22, FDR p = 0.006), and SERT (r = 0.38, FDR p < 0.001) feature maps (Supplementary Information, Fig. S4).

## Discussion

Our results indicate that the spatial pattern of vertex-wise CT alterations in the group of AN patients is associated with chemoarchitectural features of the brain. Moreover, at the individual level, the link between CT alterations and chemoarchitectural features correlated with clinical characteristics, and predicted early treatment outcome. More specifically, on the group level areas in which CT was comparatively preserved (i.e. no cortical thinning) in acutely underweight patients with AN were also characterized by higher HT1a receptor and SERT density. In contrast, those areas exhibiting higher AChN receptor density or higher glucose metabolism uptake were characterized by substantial reductions in CT in the AN group. The same analysis repeated in an external sample confirmed that the distribution of group level CT alterations in AN may align with reference chemoarchitecture features. In particular, this out-of-sample replication showed convergent evidence for preserved CT in regions with a higher density of SERT, and selective reduction in areas with a higher density of AChN.

HT1a receptors have been associated with neuronal migration, neurite outgrowth and synapse formation in the context of neurodevelopmental processes [52, 53]. Previous PET studies have shown increased HT1a binding in fronto-temporo-parietal regions, for both acutely underweight patients with AN and weight-restored individuals with a history of AN [34, 36, 37]. Interestingly, our group level analyses showed that CT in acutely underweight AN was comparatively preserved in several regions (inferior frontal gyrus, anterior temporal lobe and cingulate gyrus) previously identified as having a high density of HT1a receptors [11]. We therefore postulate that regions with a high density of HT1a receptors may benefit from neuroprotective mechanisms that counteract cortical thinning in these patients [6, 7].

Similarly, SERT expression may facilitate dendritic spine growth [54]. The neuroprotective nature of serotonin-mediated signal transduction [52, 54] may thus contribute to the finding that regions with higher cortical density of these chemoarchitecture features were also comparatively unaffected by CT reduction in in acutely underweight AN [55–57]. The selective preservation of cortical regions with a higher density of SERT (i.e. most notably in bilateral cingulate cortex, precuneus and medial occipital regions) was observed both in the main results and in the replication analysis using an external sample, thereby strengthening the reliability of this finding.

By contrast, AChN receptor density was positively associated with the pattern of CT alterations in acutely underweight patients with AN (most notably, at the group level, in midline frontoparietal regions, bilateral temporal pole, right frontolateral regions, right angular gyrus and bilateral latero-occipital regions across both samples). This result may be interpreted in light of previous research showing that nicotinic receptors are involved in appetite regulation and food intake [58], and in the development of maladaptive eating habits in rodent models of AN [32]. Notably, pharmacological activation of nicotinic receptors decreases food intake in mice, directly linking this transmitter system to food intake and weight regulation [59]. Moreover, recent evidence in animal models [33] and patients [60] shows that using donepezil to increase acetylcholine may help restore dietary behaviors. Furthermore, findings by Pena [61] suggest that AChN receptors play a critical role in postural orthostatic hypotension, a common phenomenon in AN that is often associated with energy dysregulation [62]. Given the role of nicotinic acetylcholine pathways in the central and autonomic nervous systems, it is plausible that altered AChN function may contribute to cardiovascular symptoms in AN.

The observed group level associations between CT alterations in AN and glucose uptake maps may broadly reflect low energy availability in the underweight state. They may also indicate that regions with a higher metabolic demand are those that are more vulnerable to energy restraint [60], consistent with prior findings. Specifically, cortical thinning in AN has been shown to be stronger in regions with increased energy demands using a virtual histology approach [6, 7], and most previous PET studies show that cerebral glucose metabolism is generally reduced in patients with AN [63, 64]. Further, the present associations between CT reductions and glucose uptake maps show an overlap with cognitive control and default mode networks [60], possibly reflecting behavioral alterations in decision making often observed in AN [65, 66].

Individual-level enriched CT reductions within HT1a and SERT regions were found to be significantly and moderately correlated with clinical symptom scores (i.e., depression, body dissatisfaction). The biological relevance of these individual-level enriched CT alterations can be interpreted in light of a standard theoretical model, which posits neuroplasticity and psychiatric vulnerability as two diverging presentations of a common feature at the molecular biological and cellular level [67–69]. In other words, individuals more prone to CT reductions in specific brain regions (here, regions associated with HT1a and SERT) could also exhibit a higher vulnerability to psychiatric disorders. In fact, while CT in HT1a and SERT regions was comparatively preserved at the group-level in acutely underweight patients with AN, and while SERT regions were also observed as preserved in the external sample validation, individuals who nonetheless showed CT reductions in these (commonly preserved) brain regions reported higher depressive symptoms (for HT1a), and somewhat higher body dissatisfaction (for SERT). Of note, a previous PET study revealed that there were no significant differences in SERT binding potential between a group of participants recovered from AN and HCs [38], suggesting that the distribution of SERT may be unaltered in AN. Interestingly, however, there is some previous evidence supporting possible associations between SERT polymorphisms and increased body dissatisfaction in healthy women [70, 71]. Finally, in animal models, loss of SERT function has been associated with visceral hypersensitivity [72], a condition commonly reported in patients with AN [73]. This hypersensitivity may complicate weight restoration efforts in AN by reinforcing visceral conditioning [74].

Individual-level CT alterations in areas expressing SERT also predicted early weight gain during intensive weight restoration treatment, further suggesting a relationship between structural alterations and clinical features, with potential therapeutic relevance. Again (and in line with the findings regarding higher symptom levels described above), patients with CT reductions in SERT-associated brain regions, which were otherwise comparatively preserved at the group level, gained less weight after 30 days (even after controlling for intake BMI-SDS). Higher early weight gain is a known predictor of favorable long-term outcome [16, 17, 75]. The identified neuroimaging-based SERT-associated biological signature may therefore be clinically relevant for the identification of patients experiencing higher psychological distress and at a higher risk for lower response to early weight rehabilitation. Such early identification could enable the assignment of these patients to more intensive or tailored treatment programs — for example, psychotherapeutic interventions targeting depressive symptoms [76] and body dissatisfaction, for instance behavioral therapy focused on perfectionism or self-esteem [77, 78].

## Limitations

Chemoarchitectural features (neurotransmitter receptors, transporters, and glucose metabolism) were retrieved from PET maps estimated from data in healthy individuals (mostly adults). Moreover, previously collected PET maps were not age or sex-matched to the current sample, which limits the generalizability of our findings. Consequently, results may only be tentatively interpreted as CT alterations being shaped by the underlying chemoarchitecture of the brain and causality cannot be assumed from the present results alone. Moreover, the current results were obtained in a sample of relatively young female patients diagnosed with AN, predominantly of the restrictive subtype. Consequently, the results cannot be generalized to male or older patients. Last but not least, although controls were age-matched, and age was included as a covariate in all analyses, it was not possible to fully exclude neurodevelopmental effects biasing the current results.

## Conclusions

The current study highlights the interplay between CT alterations and chemoarchitecture in the acute phase of underweight and severe food restriction in AN. Regions with more pronounced cortical thinning in AN were positively associated with a higher AChN density and higher glucose metabolic uptake. Conversely, CT in regions with higher density of HT1a receptors and SERT was comparatively preserved in AN. Out of sample replication confirmed results pertaining to AChN (positive association with cortical thinning) and SERT (higher density associated with preserved CT). Nonetheless, although HT1a receptors and SERT-associated regions were preserved on average (on the group level), if an individual patient exhibited cortical thinning in these respective regions, the same patient also reported higher psychopathology, and less early weight gain during intensive nutritional rehabilitation. These findings provide insight into a biological risk signature of AN that may aid in the identification of patients at risk of worse clinical outcomes. The current results may support future tailored approaches to treatment of AN, potentially improving outcomes in this severe medical condition.

## Supplementary information

Supplemental Material

## Data Availability

The datasets generated during the current study, as well as the code supporting the analyses, are available from the corresponding author upon reasonable request.

## References

[1] American Psychiatric Association. Diagnostic and statistical manual of mental disorders (5th ed., text rev.) 2022.

[2] Bulik CM. From awareness to action: an urgent call to address the inadequacy of treatment for anorexia nervosa. AJP. 2021;178:786–8. 10.1176/appi.ajp.2021.21070697 34516232

[3] Steinhausen HC. Outcome of eating disorders. Child Adolesc Psychiatr Clin North Am. 2009;18:225–42. 10.1016/j.chc.2008.07.013 19014869

[4] Castelpietra G, Knudsen AKS, Agardh EE, Armocida B, Beghi M, Iburg KM, et al. The burden of mental disorders, substance use disorders and self-harm among young people in Europe, 1990-2019: findings from the global burden of disease study 2019. Lancet Reg Health Eur. 2022;16:100341. 35392452 10.1016/j.lanepe.2022.100341 PMC8980870

[5] King JA, Frank GKW, Thompson PM, Ehrlich S. Structural neuroimaging of anorexia nervosa: future directions in the quest for mechanisms underlying dynamic alterations. Biol Psychiatry. 2018;83:224–34. 28967386 10.1016/j.biopsych.2017.08.011 PMC6053269

[6] Walton E, Bernardoni F, Batury V-L, Bahnsen K, Larivière S, Abbate-Daga G, et al. Brain structure in acutely underweight and partially weight-restored individuals with anorexia nervosa-a coordinated analysis by the ENIGMA eating disorders working group. Biol Psychiatry. 2022;92:730–8. 36031441 10.1016/j.biopsych.2022.04.022 PMC12145862

[7] Bahnsen K, Bernardoni F, King JA, Geisler D, Weidner K, Roessner V, et al. Dynamic structural brain changes in anorexia nervosa: a replication study, mega-analysis, and virtual histology approach. J Am Acad Child Adolesc Psychiatry. 2022;61:1168–81. 35390458 10.1016/j.jaac.2022.03.026

[8] Bernardoni F, King JA, Geisler D, Stein E, Jaite C, Nätsch D, et al. Weight restoration therapy rapidly reverses cortical thinning in anorexia nervosa: a longitudinal study. NeuroImage. 2016;130:214–22. 26876474 10.1016/j.neuroimage.2016.02.003

[9] Bomba M, Riva A, Morzenti S, Grimaldi M, Neri F, Nacinovich R. Global and regional brain volumes normalization in weight-recovered adolescents with anorexia nervosa: preliminary findings of a longitudinal voxel-based morphometry study. Neuropsychiatric Dis Treat. 2015;11:637–45. 10.2147/NDT.S73239 PMC4358418 25834442

[10] Hansen JY, Shafiei G, Markello RD, Smart K, Cox SML, Nørgaard M, et al. Mapping neurotransmitter systems to the structural and functional organization of the human neocortex. Nat Neurosci. 2022;25:1569–81. 36303070 10.1038/s41593-022-01186-3 PMC9630096

[11] Markello RD, Hansen JY, Liu Z-Q, Bazinet V, Shafiei G, Suárez LE, et al. neuromaps: structural and functional interpretation of brain maps. Nat Methods. 2022;19:1472–9. 36203018 10.1038/s41592-022-01625-w PMC9636018

[12] Castellini G, Cassioli E, Rossi E, Innocenti M, Gironi V, Sanfilippo G, et al. The impact of COVID-19 epidemic on eating disorders: a longitudinal observation of pre versus post psychopathological features in a sample of patients with eating disorders and a group of healthy controls. Int J Eat Disord. 2020;53:1855–62. 32856333 10.1002/eat.23368 PMC7461528

[13] Castellini G, Cassioli E, Rossi E, Marchesoni G, Cerini G, Pastore E, et al. Use and misuse of the emergency room by patients with eating disorders in a matched-cohort analysis: what can we learn from it? Psychiatry Res. 2023;328:115427. 37647700 10.1016/j.psychres.2023.115427

[14] Schmidt UH, Claudino A, Fernández‐Aranda F, Giel KE, Griffiths J, Hay PJ, et al. The current clinical approach to feeding and eating disorders aimed to increase personalization of management. World Psychiatry. 2025;24:4–31. 39810680 10.1002/wps.21263 PMC11733474

[15] Arold D, Bernardoni F, Geisler D, Doose A, Uen V, Boehm I, et al. Predicting long-term outcome in anorexia nervosa: a machine learning analysis of brain structure at different stages of weight recovery. Psychol Med. 2023;53:7827–36. 37554008 10.1017/S0033291723001861 PMC10758339

[16] Boehm I, Finke B, Tam FI, Fittig E, Scholz M, Gantchev K, et al. Effects of perceptual body image distortion and early weight gain on long-term outcome of adolescent anorexia nervosa. Eur Child Adolesc Psychiatry. 2016;25:1319–26. 27154049 10.1007/s00787-016-0854-1

[17] Le Grange D, Accurso EC, Lock J, Agras S, Bryson SW. Early weight gain predicts outcome in two treatments for adolescent anorexia nervosa. Intl J Eat Disord. 2014;47:124–9. 10.1002/eat.22221 PMC4341963 24190844

[18] Hemmelmann C, Brose S, Vens M, Hebebrand J, Ziegler A. [Percentiles of body mass index of 18-80-year-old German adults based on data from the second national nutrition survey]. Dtsch Med Wochenschr. 2010;135:848–52. 20408102 10.1055/s-0030-1253666

[19] Kromeyer-Hauschild K, Wabitsch M, Kunze D, Geller F, Geiß HC, Hesse V, et al. Perzentile für den body-mass-index für das kindes- und jugendalter unter heranziehung verschiedener deutscher stichproben. Monatsschr Kinderheilkd. 2001;149:807–18.

[20] Fichter M, Quadflieg N. Das strukturierte interview für anorektische und bulimische ess-störungen nach DSM-IV und ICD-10 zur expertenbeurteilung (SIAB-EX) und dazugehöriger fragebogen zur selbsteinschätzung (SIAB-S). Verhaltenstherapie. 2001;11:314–25.

[21] Paul T, Thiel A Eating Disorder Inventory-2 (EDI-2): deutsche Version. Göttingen: Hogrefe; 2005.

[22] Hautzinger M, Keller F, Kühner C. BDI-II. beck-depressions-inventar. revision 2. Frankfurt: Pearson Assessment and Information GmbH. 2009.

[23] Ehrlich S, Geisler D, Ritschel F, King JA, Seidel M, Boehm I, et al. Elevated cognitive control over reward processing in recovered female patients with anorexia nervosa. J Psychiatry Neurosci. 2015;40:307–15. 26107161 10.1503/jpn.140249 PMC4543093

[24] Hellerhoff I, King JA, Tam FI, Pauligk S, Seidel M, Geisler D, et al. Differential longitudinal changes of neuronal and glial damage markers in anorexia nervosa after partial weight restoration. Transl Psychiatry. 2021;11:86. 33558486 10.1038/s41398-021-01209-w PMC7870648

[25] Harris PA, Taylor R, Thielke R, Payne J, Gonzalez N, Conde JG. Research electronic data capture (REDCap)—a metadata-driven methodology and workflow process for providing translational research informatics support. J Biomed Inform. 2009;42:377–81. 18929686 10.1016/j.jbi.2008.08.010 PMC2700030

[26] Horndasch S, Rösch J, Kratz O, Vogel A, Heinrich H, Graap H, et al. Neural mechanisms of perceptive and affective processing of body stimuli in anorexia nervosa – are there developmental effects? Psychiatry Res. 2020;286:112853. 32114206 10.1016/j.psychres.2020.112853

[27] Horndasch S, Roesch J, Forster C, Dörfler A, Lindsiepe S, Heinrich H, et al. Neural processing of food and emotional stimuli in adolescent and adult anorexia nervosa patients. PLOS ONE. 2018;13:e0191059. 29579064 10.1371/journal.pone.0191059 PMC5868769

[28] Trefler A, Sadeghi N, Thomas AG, Pierpaoli C, Baker CI, Thomas C. Impact of time-of-day on brain morphometric measures derived from T1-weighted magnetic resonance imaging. NeuroImage. 2016;133:41–52. 26921714 10.1016/j.neuroimage.2016.02.034 PMC5602560

[29] Fischl B. FreeSurfer. NeuroImage. 2012;62:774–81. 10.1016/j.neuroimage.2012.01.021 PMC3685476 22248573

[30] Koithan EM, King JA, Ehrlich S, Haynos AF. Neuroimaging and eating disorders. In: Robinson P, Wade T, Herpertz-Dahlmann B, Fernandez-Aranda F, Treasure J, Wonderlich S, editors. Eating Disorders. Cham: Springer International Publishing; 2023. p. 1–23.

[31] Di Gianni A, De Donatis D, Valente S, De Ronchi D, Atti AR. Eating disorders: do PET and SPECT have a role? a systematic review of the literature. Psychiatry Res: Neuroimaging. 2020;300:111065. 32234640 10.1016/j.pscychresns.2020.111065

[32] Favier M, Janickova H, Justo D, Kljakic O, Runtz L, Natsheh JY, et al. Cholinergic dysfunction in the dorsal striatum promotes habit formation and maladaptive eating. J Clin Invest. 2020;130:6616–30. 33164988 10.1172/JCI138532 PMC7685731

[33] Favier M, Martin Garcia E, Icick R, de Almeida C, Jehl J, Desplanque M, et al. The human VGLUT3-pT8I mutation elicits uneven striatal DA signaling, food or drug maladaptive consumption in male mice. Nat Commun. 2024;15:5691. 38971801 10.1038/s41467-024-49371-1 PMC11227582

[34] Galusca B, Costes N, Zito NG, Peyron R, Bossu C, Lang F, et al. Organic background of restrictive-type anorexia nervosa suggested by increased serotonin1A receptor binding in right frontotemporal cortex of both lean and recovered patients: [18F]MPPF PET Scan Study. Biol Psychiatry. 2008;64:1009–13. 18639866 10.1016/j.biopsych.2008.06.006

[35] Galusca B, Traverse B, Costes N, Massoubre C, Le Bars D, Estour B, et al. Decreased cerebral opioid receptors availability related to hormonal and psychometric profile in restrictive-type anorexia nervosa. Psychoneuroendocrinology. 2020;118:104711. 32460196 10.1016/j.psyneuen.2020.104711

[36] Bailer UF, Frank GK, Henry SE, Price JC, Meltzer CC, Weissfeld L, et al. Altered brain serotonin 5-HT1A receptor binding after recovery from anorexia nervosa measured by positron emission tomography and [carbonyl11C]WAY-100635. Arch Gen Psychiatry. 2005;62:1032–41. 16143735 10.1001/archpsyc.62.9.1032

[37] Bailer UF, Frank GK, Henry SE, Price JC, Meltzer CC, Mathis CA, et al. Exaggerated 5-HT1A but normal 5-HT2A receptor activity in individuals ill with anorexia nervosa. Biol Psychiatry. 2007;61:1090–9. 17241616 10.1016/j.biopsych.2006.07.018

[38] Bailer UF, Frank GK, Henry SE, Price JC, Meltzer CC, Becker C, et al. Serotonin transporter binding after recovery from eating disorders. Psychopharmacology. 2007;195:315–24. 17690869 10.1007/s00213-007-0896-7

[39] Martin-Garcia E, Domingo-Rodriguez L, Lutz B, Maldonado R, Ruiz de Azua I. Cannabinoid type-1 receptors in CaMKII neurons drive impulsivity in pathological eating behavior. Mol Metab. 2025;92:102096. 39788291 10.1016/j.molmet.2025.102096 PMC11787564

[40] Lotter LD, Saberi A, Hansen JY, Misic B, Paquola C, Barker GJ, et al. Regional patterns of human cortex development correlate with underlying neurobiology. Nat Commun. 2024;15:7987. 39284858 10.1038/s41467-024-52366-7 PMC11405413

[41] Wiesman AI, da Silva, Castanheira J, Fon EA, Baillet S, Group P-AR, et al. Alterations of cortical structure and neurophysiology in parkinson’s disease are aligned with neurochemical systems. Ann Neurol. 2024;95:802–16. 38146745 10.1002/ana.26871 PMC11023768

[42] Buckner RL, Krienen FM, Castellanos A, Diaz JC, Yeo BTT. The organization of the human cerebellum estimated by intrinsic functional connectivity. J Neurophysiol. 2011;106:2322–45. 21795627 10.1152/jn.00339.2011 PMC3214121

[43] Robinson EC, Jbabdi S, Glasser MF, Andersson J, Burgess GC, Harms MP, et al. MSM: a new flexible framework for multimodal surface matching. NeuroImage. 2014;100:414–26. 24939340 10.1016/j.neuroimage.2014.05.069 PMC4190319

[44] Robinson EC, Garcia K, Glasser MF, Chen Z, Coalson TS, Makropoulos A, et al. Multimodal surface matching with higher-order smoothness constraints. NeuroImage. 2018;167:453–65. 29100940 10.1016/j.neuroimage.2017.10.037 PMC5991912

[45] Wu J, Ngo GH, Greve D, Li J, He T, Fischl B, et al. Accurate nonlinear mapping between MNI volumetric and FreeSurfer surface coordinate systems. Hum Brain Mapp. 2018;39:3793–808. 29770530 10.1002/hbm.24213 PMC6239990

[46] Alexander-Bloch A, Giedd JN, Bullmore E. Imaging structural co-variance between human brain regions. Nat Rev Neurosci. 2013;14:322–36. 23531697 10.1038/nrn3465 PMC4043276

[47] Benjamini Y, Hochberg Y. Controlling the false discovery rate: a practical and powerful approach to multiple testing. J Roy Stat Soc Ser B. 1995;57:289–300.

[48] Lebow J, Sim L, Crosby RD, Goldschmidt AB, Le Grange D, Accurso EC. Weight gain trajectories during outpatient family-based treatment for adolescents with anorexia nervosa. Int J Eat Disord. 2019;52:88–94. 10.1002/eat.23000 PMC7463109 30578648

[49] Bauer DJ, Curran PJ. Probing interactions in fixed and multilevel regression: inferential and graphical techniques. Multivar Behav Res. 2005;40:373–400. 10.1207/s15327906mbr4003_5 26794689

[50] Johnson PO, Fay LC. The Johnson-Neyman technique, its theory and application. Psychometrika. 1950;15:349–67. 14797902 10.1007/BF02288864

[51] Long JS, Ervin LH. Using heteroscedasticity consistent standard errors in the linear regression model. Am Statistician. 2000;54:217–24.

[52] Chilmonczyk Z, Bojarski AJ, Pilc A, Sylte I. Serotonin transporter and receptor ligands with antidepressant activity as neuroprotective and proapoptotic agents. Pharmacol Rep. 2017;69:469–78. 28324844 10.1016/j.pharep.2017.01.011

[53] Marco I, Valhondo M, Martín-Fontecha M, Vázquez-Villa H, Del Río J, Planas A, et al. New serotonin 5-HT _1A_ receptor agonists with neuroprotective effect against ischemic cell damage. J Med Chem. 2011;54:7986–99. 22029386 10.1021/jm2007886

[54] Chaji D, Venkatesh VS, Shirao T, Day DJ, Ellenbroek BA. Genetic knockout of the serotonin reuptake transporter results in the reduction of dendritic spines in in vitro rat cortical neuronal culture. J Mol Neurosci. 2021;71:2210–8. 33403594 10.1007/s12031-020-01764-9

[55] Abou Al Hassan S, Cutinha D, Mattar L. The impact of COMT, BDNF and 5-HTT brain-genes on the development of anorexia nervosa: a systematic review. Eat Weight Disord-Studies Anorexia, Bulim Obes. 2021;26:1323–44. 10.1007/s40519-020-00978-5 32783113

[56] Calati R, De Ronchi D, Bellini M, Serretti A. The 5‐HTTLPR polymorphism and eating disorders: a meta‐analysis. Intl J Eat Disord. 2011;44:191–9. 10.1002/eat.20811 20209488

[57] Lee Y, Lin P. Association between serotonin transporter gene polymorphism and eating disorders: a meta‐analytic study. Intl J Eat Disord. 2010;43:498–504. 10.1002/eat.20732 19708070

[58] Jo Y, Talmage DA, Role LW. Nicotinic receptor‐mediated effects on appetite and food intake. J Neurobiol. 2002;53:618–32. 12436425 10.1002/neu.10147 PMC2367209

[59] Mineur YS, Abizaid A, Rao Y, Salas R, DiLeone RJ, Gündisch D, et al. Nicotine decreases food intake through activation of POMC neurons. Science. 2011;332:1330–2. 21659607 10.1126/science.1201889 PMC3113664

[60] Friars D, Walsh O, McNicholas F. Assessment and management of cardiovascular complications in eating disorders. J Eat Disord. 2023;11:1–12. 36717950 10.1186/s40337-022-00724-5 PMC9886215

[61] Vaishnavi SN, Vlassenko AG, Rundle MM, Snyder AZ, Mintun MA, Raichle ME. Regional aerobic glycolysis in the human brain. Proc Natl Acad Sci. 2010;107:17757–62. 20837536 10.1073/pnas.1010459107 PMC2955101

[62] Pena C, Moustafa A, Mohamed A-R, Grubb B. Autoimmunity in syndromes of orthostatic intolerance: an updated review. J Pers Med. 2024;14:435. 38673062 10.3390/jpm14040435 PMC11051445

[63] Delvenne V, Lotstra F, Goldman S, Biver F, De Maertelaer V, Appelboom-Fondu J, et al. Brain hypometabolism of glucose in anorexia nervosa: a PET scan study. Biol Psychiatry. 1995;37:161–9. 7727624 10.1016/0006-3223(94)00189-A

[64] van Waarde A, Audenaert K, Busatto GF, Buchpiguel C, Dierckx RAJO. SPECT and PET in Eating Disorders. In: Dierckx RAJO, Otte A, de Vries EFJ, van Waarde A, Sommer IE, editors. PET and SPECT in Psychiatry. Cham: Springer International Publishing; 2021. p. 741–72.

[65] Doose A, King JA, Bernardoni F, Geisler D, Hellerhoff I, Weinert T, et al. Strengthened default mode network activation during delay discounting in adolescents with anorexia nervosa after partial weight restoration: a longitudinal fMRI study. J Clin Med. 2020;9:900. 32218141 10.3390/jcm9040900 PMC7230250

[66] Guillaume S, Gorwood P, Jollant F, Van den Eynde F, Courtet P, Richard-Devantoy S. Impaired decision-making in symptomatic anorexia and bulimia nervosa patients: a meta-analysis. Psychological Med. 2015;45:3377–91. 10.1017/S003329171500152X 26497047

[67] Branchi I. The double edged sword of neural plasticity: increasing serotonin levels leads to both greater vulnerability to depression and improved capacity to recover. Psychoneuroendocrinology. 2011;36:339–51. 20875703 10.1016/j.psyneuen.2010.08.011

[68] Hettwer MD, Larivière S, Park BY, van den Heuvel OA, Schmaal L, Andreassen OA, et al. Coordinated cortical thickness alterations across six neurodevelopmental and psychiatric disorders. Nat Commun. 2022;13:6851. 36369423 10.1038/s41467-022-34367-6 PMC9652311

[69] Paus T, Keshavan M, Giedd JN. Why do many psychiatric disorders emerge during adolescence? Nat Rev Neurosci. 2008;9:947–57. 19002191 10.1038/nrn2513 PMC2762785

[70] Frieling H, Römer KD, Wilhelm J, Hillemacher T, Kornhuber J, de Zwaan M, et al. Association of catecholamine-O-methyltransferase and 5-HTTLPR genotype with eating disorder-related behavior and attitudes in females with eating disorders. Psychiatr Genet. 2006;16:205–8. 16969275 10.1097/01.ypg.0000218620.50386.f1

[71] Castellini G, Ricca V, Lelli L, Bagnoli S, Lucenteforte E, Faravelli C, et al. Association between serotonin transporter gene polymorphism and eating disorders outcome: a 6-year follow-up study. Am J Med Genet Part B: Neuropsychiatric Genet. 2012;159B:491–500. 10.1002/ajmg.b.32052 22488946

[72] El-Ayache N, Galligan JJ. 5-HT3 receptor signaling in serotonin transporter-knockout rats: a female sex-specific animal model of visceral hypersensitivity. Am J Physiol-Gastrointestinal Liver Physiology. 2019;316:G132–G143. 10.1152/ajpgi.00131.2018 PMC6383387 30359082

[73] Salvioli B, Pellicciari A, Iero L, Di Pietro E, Moscano F, Gualandi S, et al. Audit of digestive complaints and psychopathological traits in patients with eating disorders: a prospective study. Dig Liver Dis. 2013;45:639–44. 23582347 10.1016/j.dld.2013.02.022

[74] Zucker NL, Bulik CM. On bells, saliva, and abdominal pain or discomfort: early aversive visceral conditioning and vulnerability for anorexia nervosa. Int J Eat Disord. 2020;53:508–12. 32141642 10.1002/eat.23255 PMC8344083

[75] Madden S, Miskovic‐Wheatley J, Wallis A, Kohn M, Hay P, Touyz S. Early weight gain in family‐based treatment predicts greater weight gain and remission at the end of treatment and remission at 12‐month follow‐up in adolescent anorexia nervosa. Intl J Eat Disord. 2015;48:919–22. 10.1002/eat.22414 26488111

[76] Cassioli E, Rossi E, Martelli M, Arganini F, Giuranno G, Siviglia S, et al. Longitudinal coupling between eating disorder psychopathology and depression in patients with anorexia nervosa and bulimia nervosa treated with enhanced cognitive behavior therapy: a one-year follow-up study. Brain Sci. 2023;13:535. 37190499 10.3390/brainsci13040535 PMC10136486

[77] Delaquis CP, Godart N, Barry C, Ringuenet D, Maria A-S, Nicolas I, et al. Perfectionism, self-esteem, and affective symptoms in anorexia nervosa subtypes: a network analysis of French inpatients. J Clin Psychol. 2024;80:1852–75. 38646977 10.1002/jclp.23698

[78] Kästner D, Löwe B, Weigel A, Osen B, Voderholzer U, Gumz A. Factors influencing the length of hospital stay of patients with anorexia nervosa – results of a prospective multi-center study. BMC Health Serv Res. 2018;18:22. 29334934 10.1186/s12913-017-2800-4 PMC5769422

