# Supplementary material for: Mapping the path to recovery: the intersection of cortical thickness reductions and serotonin transporter expression in anorexia nervosa
Source: Mol Psychiatry. 2025 Nov 29;31(3):1723–32. doi: 10.1038/s41380-025-03306-4 (PMC12916299; doi:10.1038/s41380-025-03306-4)
Supplement: Supplementary file 1 — Supplemental Material [file 41380_2025_3306_MOESM1_ESM.docx]

# Supplementary Information

**Methods S1 – Sample and participants**

The initial sample consisted of 120 AN and 207 HC participants of which 6 AN and 14 HC MRI scans had to be excluded due to artifacts that influence parcellation (mainly dura inclusions in the pial surface). The 114 AN participants whose data survived quality control were age-matched to HC participants using the Munkres algorithm to search for optimal pairs, resulting in a maximum age difference of 8 months between individuals within each pair.

The diagnosis of AN was made using the expert form of the Structured Interview for Anorexia and Bulimia Nervosa (SIAB-EX) [1] and required a Body Mass Index (BMI) below the 10^th^ age percentile (if < 15.5 years old) or below 17.5 kg/m² (if > 15.5 years old). Clinical experts and medical records provided information on comorbidities. HC had to be of normal weight, eumenorrheic and without any history of psychiatric illness as assessed with the M.I.N.I./M.I.N.I.-Kid [2, 3]. All HCs were recruited by advertisement among middle/high school and university students [1].

The inpatient treatment for participants with AN included a structured meal plan, developed in collaboration with a registered dietician, which was adjusted individually, taking into account dietary intake prior to admission to inpatient treatment and the severity of malnutrition.

**Methods S2 - Description of Spatial Feature Maps**

Image acquisition parameters: 176 sagittal slices (1 mm thickness, no gap, TR=1900 ms; TE=2.26 ms; flip angle=9°; voxel size=1.0×1.0×1.0 mm, FoV=256×224 mm 2, bandwidth of 200 Hz/pixel). Reconstruction of the cerebral cortex and subcortical surfaces was accomplished automatically with FreeSurfer 7.1.1, [4, 5] followed by standardized quality control by trained raters, as described in previous works [6, 7].

CT was defined as the shortest distance between the pial surface and the gray-white matter boundary. CT was then calculated for each vertex on the tessellated pial surface. In accordance with previous studies, a Gaussian smoothing kernel with a full-width-at-half-maximum (FWHM) of 10 mm was employed in order to smooth whole-brain CT [7, 8]. To calculate global CT from the mean CT of both hemispheres, we used the surface area of each hemisphere as a weighting factor.

**Methods S3 - Description of Spatial Feature Maps**

The reference map of the surface distribution of AChN receptors was derived from PET binding scans using [^18^F]Flubatine as the radioactive tracer of choice, among 30 healthy participants [9]. AChM1 was assessed in 11 healthy participants using the PET radiotracer ^11^C-LSN3172176 [10]. DAT was assessed through DAT-SPECT in six elderly participants [11]. The D1 receptor surface distribution map was estimated using D1R-selective radiotracer [^11^C]SCH23390 in PET scans of thirteen healthy volunteers [12]. The spatial distribution of D2 receptors on the cortical surface was assessed by [^18^F]Fallypride binding at PET among 49 healthy participants [13]. The expression of serotonin receptors (HT1a, HT1b, HT2a) was estimated by [^11^C]CUMI-101, [^11^C]AZ10419369, and [^11^C]Cimbi-36 binding at PET, among healthy participants (8, 36 and 29 volunteers, respectively) [14]. SERT distribution was assessed by PET tracer binding of [^11^C]MADAM among six young male subjects (age range 21-35 years old) and 10 older individuals (8 males and 2 females, age range 51-67 years old) [15]. NET binding was evaluated by using the PET tracer [^11^C]Methyl-Reboxetine, across 77 healthy participants [16, 17]. H3 distribution was estimated by PET binding of radioligand [^11^C]GSK189254 in eight healthy volunteers. MOR [18], CB1 [19], GABA [20], and glutamate were evaluated by PET binding of radioligands [11C]carfentanil, [^18^F]FMPEP-d2, [^11^C]flumazenil, and [^11^C]ABP688 (in 204, 22, 6, 28 healthy volunteers, respectively). Glucose metabolism was evaluated using [18F]-labeled fluorodeoxyglucose and assessing task-induced aerobic glycolysis in 33 healthy volunteers [21]. Please refer to the original studies for further information about the reference maps included.

**Methods S4 – Replication analysis methods**

3D T1-weighted brain volumetric MP-RAGE data was acquired in Erlangen using a 3T Siemens Trio MRI scanner (TR = 1900 ms, TE = 2.25 ms, flip angle = 9°, FOV = 256×256×256 mm, 1x1x1mm isotropic). Structural MRI was processed using all steps included in FreeSurfer *recon-all*. The ENIGMA protocol for quality assurance was performed at each site prior to analysis (http://enigma.usc.edu/protocols/imaging-protocols).The Ethics Committee of the University Hospital of Erlangen gave approval for the study, and it was conducted in accordance with the Declaration of Helsinki (220_15B).

**Table S1 - Sample descriptives of replication analysis**

|  | **AN** | **HC** | **T-score** | **p-value** | |
| --- | --- | --- | --- | --- | --- |
|  | (n = 21) | (n = 28) |  |  | |
| Demographics |  |  |  |  | |
| Age  (years ± SD) | 22.2 ± 7.75.6 | 20.9 ± 5.6 | -0.7 | 0.513 | |
| BMI | 14.8 ± 1.1 | 21.8 ± 2.4 | 13.4 | <0.001 | |
| BMI-SDS | -3,8 ± 1.3 | 0.0 ± 0.8 | 12.1 | <0.001 | |
| Note: Restrictive subtype was diagnosed in 14 AN (66.60%) and binge/purge subtype in 5 (23.81%). Mean value ± standard deviation (SD) for each variable and study group are shown. Group differences were tested using Welch two sample t-tests. As test statistics, t-value, and p-values are stated.  Legend: AN = anorexia nervosa; HC = healthy controls | | | | |  |

| **Table S2 - Control Analyses, the effect of SERT on ΔBMI-SDS restoration** | | | | |
| --- | --- | --- | --- | --- |
|  | **Main Effect**  **(SERT)** |  | **Moderation term**  **(interaction with BMI-SDS at baseline)** |  |
| No psychiatric comorbidities | -1.943  (p = 0.009) | * | -0.653  (p = 0.006) | * |
| No recent medication with antidepressants | -1.606  (p = 0.016) | * | -0.522  (p = 0.010) | * |
| Restrictive subtype only | -1.592  (p = 0.019) | * | -0.524  (p = 0.010) | * |
| *Note*: Linear models corrected for the effect of weight and age at baseline.  Restrictive subtype was diagnosed in 102 AN (89.47%) and binge/purge subtype in 12 (10.53%). Eighteen patients had other psychiatric comorbidities. Four patients had a recent exposure to antidepressant drugs (prior 14 days), none were recently exposed to antipsychotics.  * = significant at p <0.05 | | | |  |

**Figure S1a -** Group-level differences in CT, Anorexia Nervosa (AN) vs age and sex-matched Healthy Controls (HC), in Z-scores in the main sample.


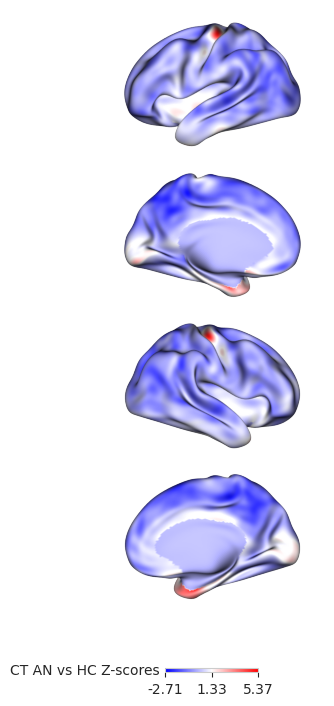


**S1b -** Group-level differences in CT, Anorexia Nervosa (AN) vs age and gender-matched Healthy Controls (HC), in Z-scores in the replication sample


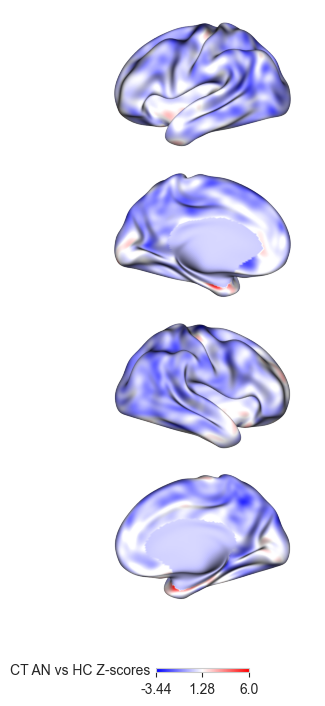


**Figure S2 - Spatial enrichment of group-level CT reduction in AN,** visual depiction of overlapping cortical areas exhibiting both CT reduction in AN and high chemoarchitecture density. Cortical overlaps driving statistical correlations were visualized by plotting the areas with the highest and lowest values in the reference feature map (in green, regions with highest values, for HT1a highest values in yellow). This overlay was performed thresholding the feature maps at 1 standard deviation (SD) from the mean (except for Glc, which was thresholded at 0.5 SD to enhance visual clarity).
*A:* AChN, Acetylcholine nicotinic receptor*; B:* HT1a, serotonin receptor; *C:* SERT, Serotonin transporter; *D:* Glc, glucose metabolism


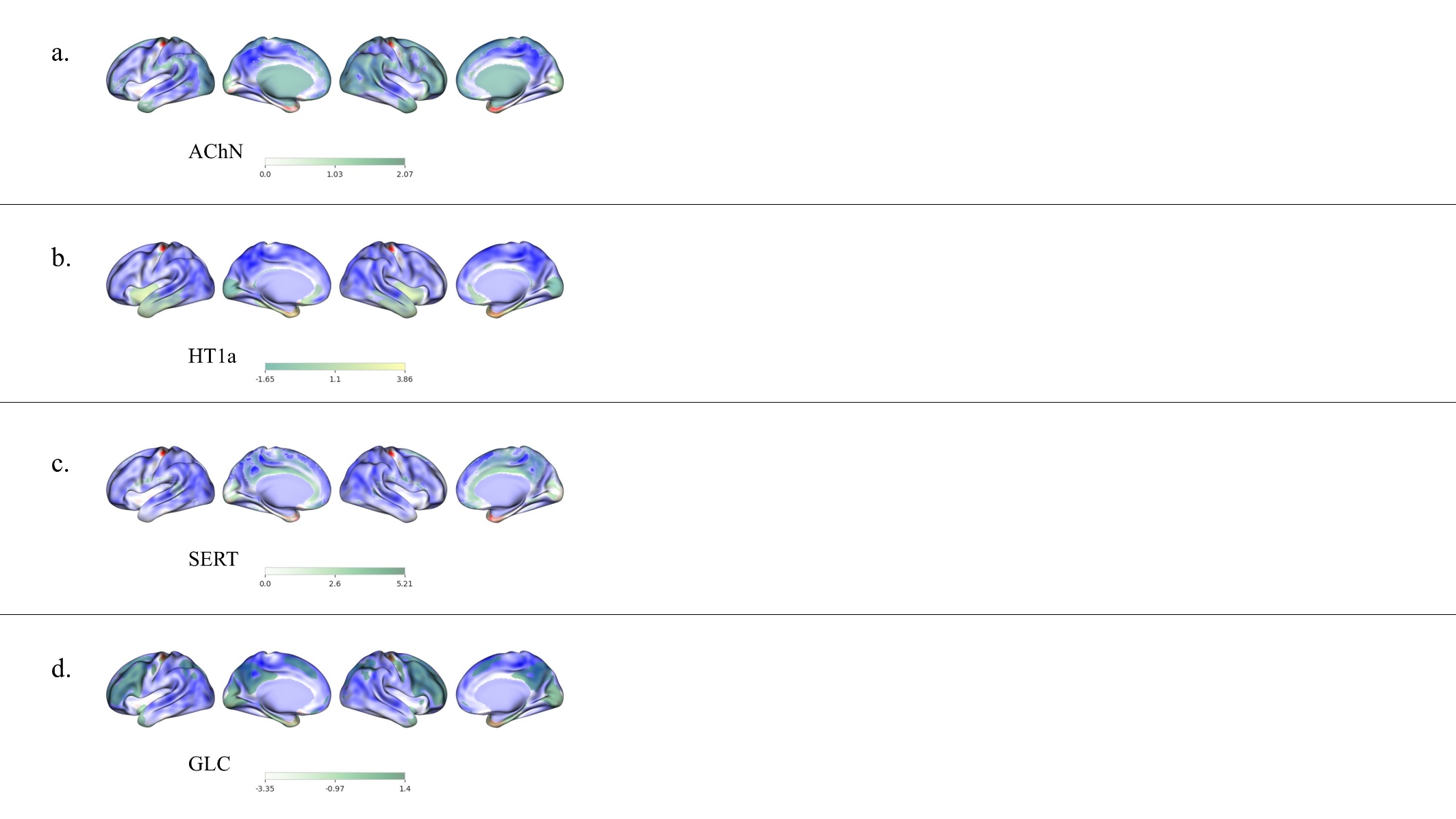


**Figure S3 -** Moderation effect of weight reduction at baseline on the effect of SERT, for the prediction of weight restoration after 30 days of intensive treatment. Individual-level CT alterations in areas expressing SERT impacted weight restoration only once a certain severity threshold was reached. Here, data was filtered to include only AN patients below -2 BMI-SDS at baseline. On the y-axis, the slope of SERT. On the x-axis, BMI-SDS.

n.s. = not significant ( p > 0.05)
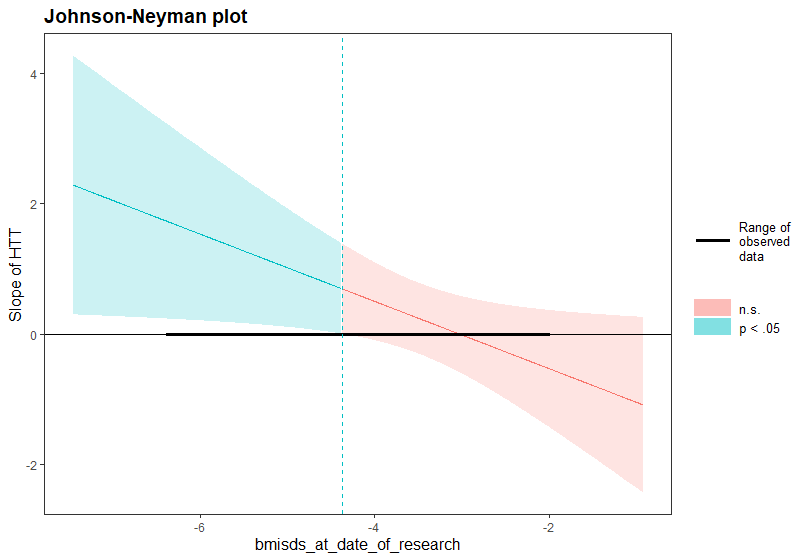


**Figure S4** - Spatial enrichment of group-level CT alterations in AN, compared to age and sex-matched healthy controls in the replication sample.


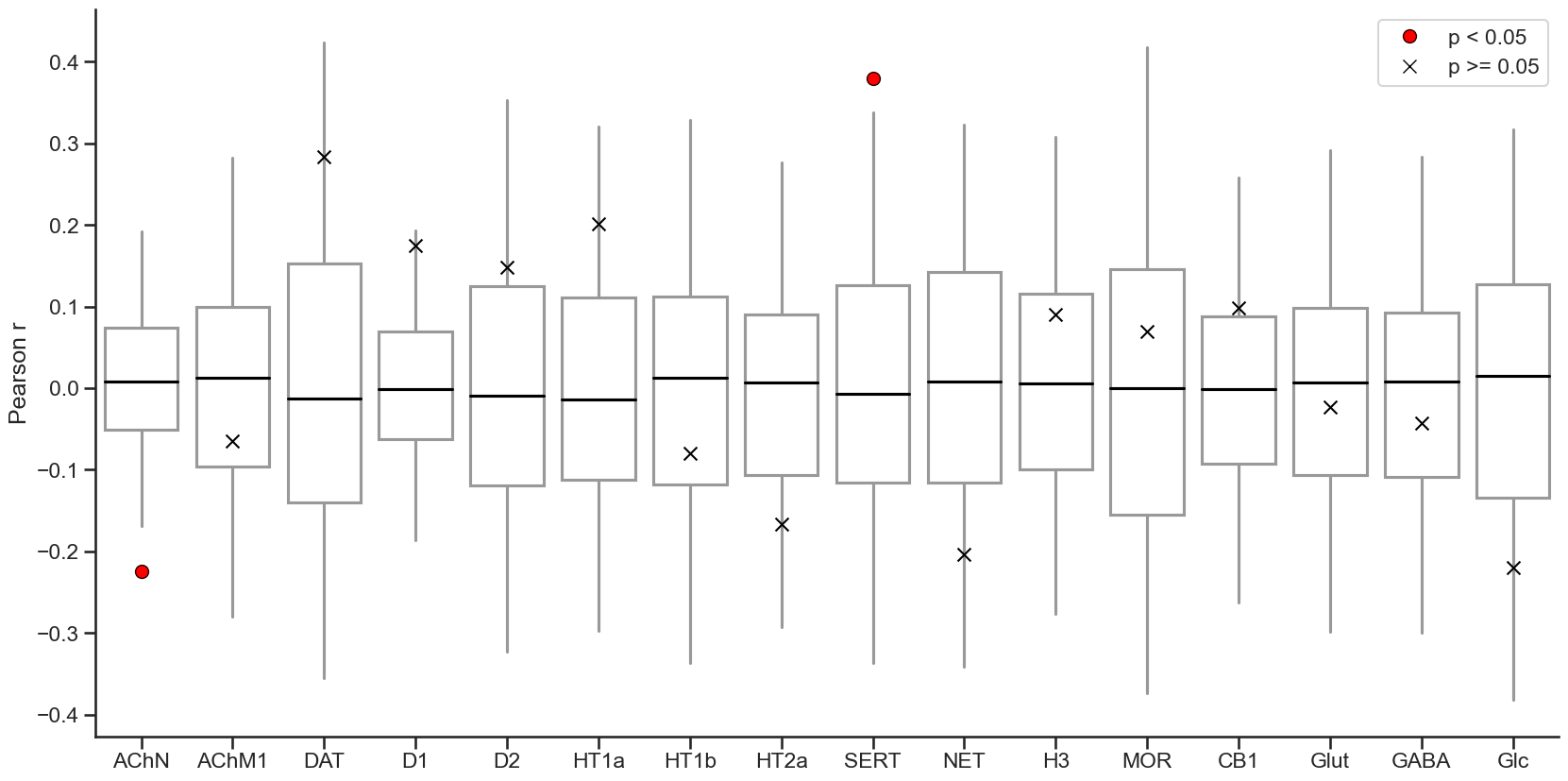


# References

1. Fichter M, Quadflieg N. Das Strukturierte Interview für Anorektische und Bulimische Ess-Störungen nach DSM-IV und ICD-10 zur Expertenbeurteilung (SIAB-EX) und dazugehöriger Fragebogen zur Selbsteinschätzung (SIAB-S). Verhaltenstherapie. 2001;11:314–325.

2. Sheehan DV, Lecrubier Y, Sheehan KH, Amorim P, Janavs J, Weiller E, et al. The Mini-International Neuropsychiatric Interview (M.I.N.I.): the development and validation of a structured diagnostic psychiatric interview for DSM-IV and ICD-10. J Clin Psychiatry. 1998;59 Suppl 20:22-33;quiz 34-57.

3. Sheehan DV, Sheehan KH, Shytle RD, Janavs J, Bannon Y, Rogers JE, et al. Reliability and validity of the Mini International Neuropsychiatric Interview for Children and Adolescents (MINI-KID). J Clin Psychiatry. 2010;71:313–326.

4. Fischl B, Salat DH, Busa E, Albert M, Dieterich M, Haselgrove C, et al. Whole Brain Segmentation: Automated Labeling of Neuroanatomical Structures in the Human Brain. Neuron. 2002;33:341–355.

5. Fischl B, Dale AM. Measuring the thickness of the human cerebral cortex from magnetic resonance images. Proceedings of the National Academy of Sciences. 2000;97:11050–11055.

6. Bahnsen K, Bernardoni F, King JA, Geisler D, Weidner K, Roessner V, et al. Dynamic Structural Brain Changes in Anorexia Nervosa: A Replication Study, Mega-analysis, and Virtual Histology Approach. Journal of the American Academy of Child & Adolescent Psychiatry. 2022;61:1168–1181.

7. Hellerhoff I, Bernardoni F, Bahnsen K, King JA, Doose A, Pauligk S, et al. Serum neurofilament light concentrations are associated with cortical thinning in anorexia nervosa. Psychological Medicine. 2023:1–9.

8. Bernardoni F, King JA, Geisler D, Stein E, Jaite C, Nätsch D, et al. Weight restoration therapy rapidly reverses cortical thinning in anorexia nervosa: A longitudinal study. NeuroImage. 2016;130:214–222.

9. Hillmer AT, Esterlis I, Gallezot JD, Bois F, Zheng MQ, Nabulsi N, et al. Imaging of cerebral α4β2* nicotinic acetylcholine receptors with (−)-[18F]Flubatine PET: Implementation of bolus plus constant infusion and sensitivity to acetylcholine in human brain. NeuroImage. 2016;141:71–80.

10. Naganawa M, Nabulsi NB, Henry S, Matuskey D, Lin S, Slieker L, et al. First in Human Assessment of the Novel M1 Muscarinic Acetylcholine Receptor PET Radiotracer 11C-LSN3172176. Journal of Nuclear Medicine. 2020. 1 August 2020. https://doi.org/10.2967/jnumed.120.246967.

11. Dukart J, Holiga Š, Chatham C, Hawkins P, Forsyth A, McMillan R, et al. Cerebral blood flow predicts differential neurotransmitter activity. Sci Rep. 2018;8:4074.

12. Kaller S, Rullmann M, Patt M, Becker G-A, Luthardt J, Girbardt J, et al. Test-retest measurements of dopamine D1-type receptors using simultaneous PET/MRI imaging. Eur J Nucl Med Mol Imaging. 2017;44:1025–1032.

13. Jaworska N, Cox SML, Tippler M, Castellanos-Ryan N, Benkelfat C, Parent S, et al. Extra-striatal D2/3 receptor availability in youth at risk for addiction. Neuropsychopharmacol. 2020;45:1498–1505.

14. Beliveau V, Ganz M, Feng L, Ozenne B, Højgaard L, Fisher PM, et al. A High-Resolution In Vivo Atlas of the Human Brain’s Serotonin System. J Neurosci. 2017;37:120–128.

15. Fazio P, Schain M, Varnäs K, Halldin C, Farde L, Varrone A. Mapping the distribution of serotonin transporter in the human brainstem with high-resolution PET: Validation using postmortem autoradiography data. NeuroImage. 2016;133:313–320.

16. Ding Y-S, Singhal T, Planeta-Wilson B, Gallezot J-D, Nabulsi N, Labaree D, et al. PET imaging of the effects of age and cocaine on the norepinephrine transporter in the human brain using (S,S)-[11C]O-methylreboxetine and HRRT. Synapse. 2010;64:30–38.

17. Hansen JY, Shafiei G, Markello RD, Smart K, Cox SML, Nørgaard M, et al. Mapping neurotransmitter systems to the structural and functional organization of the human neocortex. Nat Neurosci. 2022;25:1569–1581.

18. Kantonen T, Karjalainen T, Isojärvi J, Nuutila P, Tuisku J, Rinne J, et al. Interindividual variability and lateralization of μ-opioid receptors in the human brain. Neuroimage. 2020;217:116922.

19. Laurikainen H, Tuominen L, Tikka M, Merisaari H, Armio R-L, Sormunen E, et al. Sex difference in brain CB1 receptor availability in man. NeuroImage. 2019;184:834–842.

20. Nørgaard M, Beliveau V, Ganz M, Svarer C, Pinborg LH, Keller SH, et al. A high-resolution in vivo atlas of the human brain’s benzodiazepine binding site of GABAA receptors. NeuroImage. 2021;232:117878.

21. Vaishnavi SN, Vlassenko AG, Rundle MM, Snyder AZ, Mintun MA, Raichle ME. Regional aerobic glycolysis in the human brain. Proceedings of the National Academy of Sciences. 2010;107:17757–17762.
